# The shear band controlled deformation in metallic glass: a perspective from fracture

**DOI:** 10.1038/srep21852

**Published:** 2016-02-22

**Authors:** G. N. Yang, Y. Shao, K. F. Yao

**Affiliations:** 1 School of Material Science and Engineering, Tsinghua University, Beijing 100084, P.R. China; 2 Key Laboratory for Advanced Materials Processing Technology, Ministry of Education, P.R. China

## Abstract

Different from the homogenous deformation in conventional crystalline alloys, metallic glasses and other work-softening materials deform discontinuously by localized plastic strain in shear bands. Here by three-point bending test on a typical ductile Pd-Cu-Si metallic glass, we found that the plastic deformed region during fracture didn’t follow the yielding stress distribution as the conventional material mechanics expected. We speculated that such special behavior was because the shear bands in metallic glasses could propagate easily along local shear stress direction once nucleated. Based on a 3D notch tip stress field simulation, we considered a new fracture process in a framework of multiple shear band deformation mechanism instead of conventional materials mechanics, and successfully reproduced the as-observed complicate shear band morphologies. This work clarifies many common misunderstandings on metallic glasses fracture, and might also provide a new insight to the shear band controlled deformation. It suggests that the deformation of metallic glasses is sensitive to local stress condition, and therefore their mechanical properties would depend on not only the material, but also other external factors on stress condition. We hope that start from this work, new methods, criteria, or definitions could be proposed to further study these work-softening materials, especially for metallic glasses.

With work-hardening effect and the behavior of dislocations, the deformation of many crystalline metallic alloys will experience an elastic, yielding, work-hardening, and fracture process. Based on such deformation route, modern materials mechanics are established. However, there are still many materials in nature showing work-softening effects instead of work-hardening, including metallic glasses^1^, granular materials^2^, nanocrystalline metals^3^, complex liquids^4^ and some crystalline alloys under dynamic loading^5^ . In those materials, the stress is not uniformly distributed but rather localized in the so-called shear bands, and the deformation is realized by the shear band movement. Controlled by this shear band mechanism, the deformation will follow an elastic, shear band sliding, and fracture process, which differs from that of conventional metallic alloys. In metallic glasses, besides the localized and discontinued deformation behavior, it has also been found that these materials show scattered values in plasticity and fracture toughness. Even for the same material, the properties strongly depend on the sample size, shape, and loading condition^6^,^7^,^8^,^9^. All these facts suggest that these work-softening materials fall out of the scope of conventional materials mechanics. After many theoretical and experimental works to study the shear band from physical nature to external behavior^1^, there still is a long distance from the exact description of this shear band controlled deformation, i.e. how a material deforms via multiple shear bands. In the studies on deformation behaviors of metallic glasses, the concepts and measuring methods from conventional materials mechanics were still widely used as important references^10^,^11^,^12^,^13^. However, with deeper research, some drawbacks and principle issues have emerged. At present, to face the question of shear band controlled deformation, a primary problem is to recognize the fundamental difference between such behaviors with the conventional materials mechanics. Based on that, a more sound materials mechanics for shear band controlled deformation could be built, especially for metallic glasses.

This question has been studied from a perspective of fracture^14^, as it was not only an important engineering property, but also a good model to explore the deformation mechanism by the complex loading condition. In polycrystalline materials, the classic linear elastic fracture mechanics (LEFM) depicts a plastic zone by a contour line of yielding stress, which provides an accessible method to understand the fracture process in these materials^15^. (It should be noticed that the deformation inside the plastic zone could redistribute the stress field and lead to the result slightly different, and thus the LEFM is only a simplification) In metallic glasses, finite element method (FEM) simulation was applied to study the stress distribution and therefore predict the shear band or plastic deformed region morphologies. The results consist with experiments in nano-indentation^16^ and mixed mode crack-tip stress field^17^. However, the shear band morphology in Mode I crack-tip stress field is apparently different from the conventional plastic zone in this situation^11^. It is then concerned whether the FEM is not applicable for predicting the stress field in metallic glasses or the metallic glasses behave differently from polycrystalline alloys under the same stress field.

In this paper, we studied this question by three-point bending test on a typical ductile Pd-Cu-Si metallic glass, we found that their plastic deformed regions didn’t follow the yielding stress distribution as the conventional material mechanics expected. Different shear band morphologies also showed up at different locations of the sample. We speculated that such special behavior was because the shear bands in metallic glasses could propagate easily along local shear stress direction once nucleated. Then based on a 3D notch tip stress field simulated by FEM, we considered a new fracture process in a framework of multiple shear band deformation mechanism instead of conventional materials mechanics, and successfully reproduced the as-observed complicated shear band morphologies. The results clarified many common misunderstandings on metallic glasses fracture. It might also provide a new insight to the deformation behavior of metallic glasses and even other work-softening materials deform by shear bands. As the shear band behavior is sensitive to the local stress condition and distribution, it therefore becomes reasonable to understand the scattered and variable mechanical properties of metallic glasses, and the affection from sample size, shape, loading method and other parameters. Based on such new understanding, we suggest that those concepts in classic materials mechanics, such as yielding stress, plasticity and fracture toughness, need to be reconsidered in these work-softening materials.

## Results

### Fracture experiment of a Pd-Cu-Si metallic glass

To reveal the difference of such shear band controlled deformation from the conventional materials mechanics, we performed 3-point bending test on a notched Pd_77.5_Cu_6_Si_16.5_ bulk metallic glass (BMG), a typical ductile glassy alloy. Details of the experimental measurement are described in Methods.

**Figure 1:**
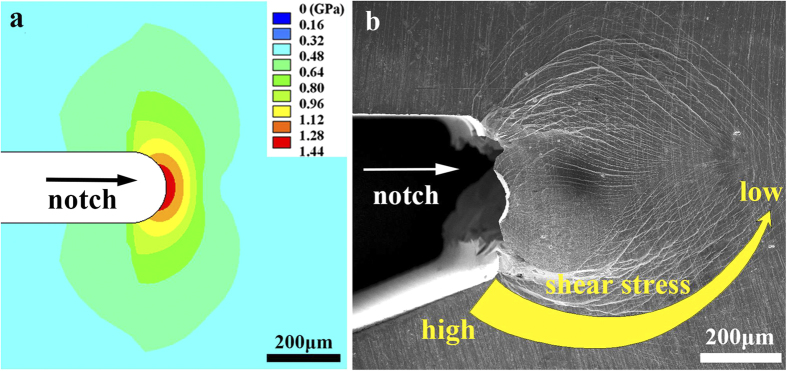
(**a**) The SEM image of the notch tip region of a Pd_77.5_Cu_6_Si_16.5_ metallic glass. (**b**) The Mohr–Coulomb stress field distribution on the sample’s side surface simulated by FEM. It shows a mismatch between the shear band morphologies and the stress distribution, and a decreasing shear stress intensity along the shear band path.

Figure 1a shows the side view of the notch tip appearance of the sample under scanning electron microscope (SEM). It shows that the sample has severely deformed with vast curved shear bands formation. In the classic LEFM for traditional polycrystalline material fracture, a plastic zone could be drawn near the notch tip by a contour line of yielding stress, where the material inside the plastic zone could deform plastically, and the material outside the zone will be still elastic. To predict the plastic zone shape in our sample, we performed a 3D notch tip stress field simulation by FEM (Details of the simulation will be discussed later in the paper and Methods section). Then we achieved a stress distribution on the sample’s side surface as shown in Fig. 1b. By comparing it with experiment (Fig. 1a), we found that the real plastic deformed region (the region with shear bands) didn’t follow the plastic zone description in LEFM. (In this paper we named it as plastic region to distinguish it from the plastic zone concept in LEFM.) It seemed that the stress on the boundary of the plastic region was not constant but would decrease along the shear band. This difference implies that the fracture and deformation mechanism in this material might not be understood from a traditional material mechanics.

**Figure 2:**
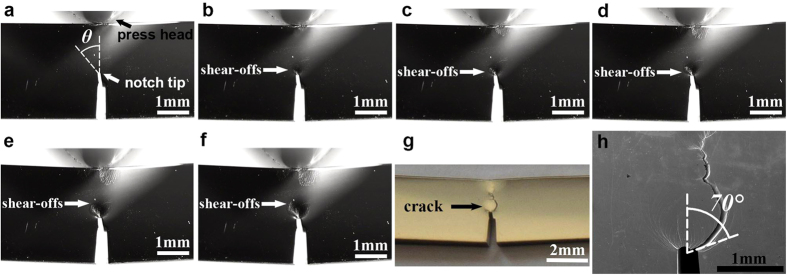
The side view of the sample during 3-point bending and after fracture. (**a**–**f**) show the evolution of shear-offsets recorded by an optical camera as the loading time increased. (**g**,**h**) show the side view of the sample after fracture, recorded by optical camera and SEM, respectively.

The evolution of these shear bands on the sample’s side surface during loading was recorded by an optical camera and shown in Fig. 2a–f. This process was more clearly shown in the video in supplement materials. It was found that these shear bands didn’t form randomly at the notch tip but came out approximately in a sequence manner from low angle (*θ*) part to high angle part with increasing length. After final fracture, the side view of the sample was recorded by optical camera and SEM, showed in Fig. 2g,h, respectively. It was found that the final fracture occurred along a later formed shear band at *θ* ≈ 70° but not the initially formed shear band at *θ* ≈ 0°.

**Figure 3:**
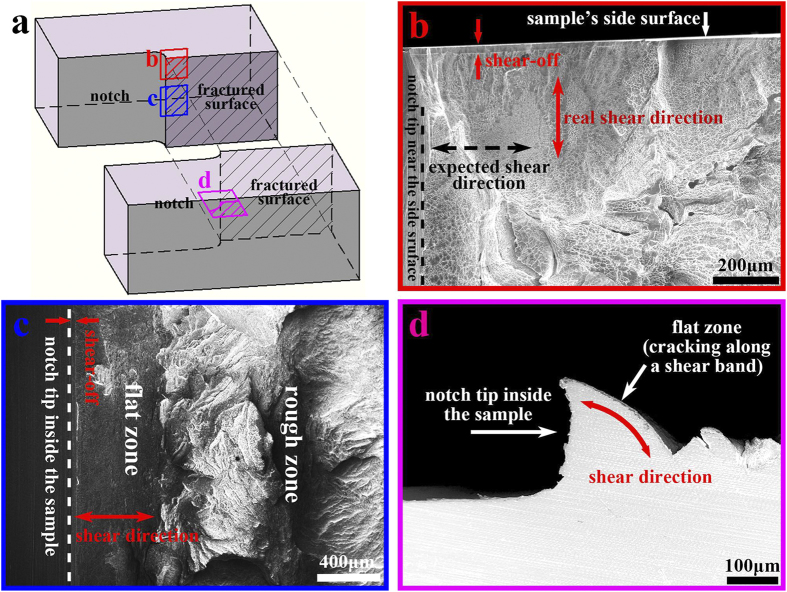
The SEM images of the fractograph of the sample. (**a**) Sketch of the fractured sample with three marked regions for SEM inspection. (**b**) The SEM image of fractograph near the side surface. (**c**) The SEM image of fractograph inside the sample. (**d**) The SEM image of cross section profile inside the sample. The fractograph showed two shear band morphologies with different shear directions marked in (**b**,**c**) at different positions of the notch.

To inspect the fracture morphology more clearly, the fractured pieces were cut from the middle so that the crack propagation path inside the sample could be explored. Figure 3 shows the SEM images of the fractograph from three different perspectives: near the sample’s side surface (Fig. 3b), inside the sample (Fig. 3c), and the cross section inside the sample (Fig. 3d). These fractographs are typical in metallic glasses fracture, formed by shear bands rupture^18^. The shear bands near the side surface have a cone shape, while the shear bands inside the sample show an approximate cylindroid shape. As the result of shear band sliding, the morphologies of shear-offsets on these two kinds of shear bands indicate two different shear directions as marked in Fig. 3.

At present there are several viewpoints to interpret the formation of these different shear band morphologies. For the shear bands inside the sample (Fig. 3c), the fractograph was recognized as a flat zone and a rough zone^19^ (or planar region and over load region^20^) according to its morphology in SEM. However, Fig. 3d shows that this flat zone is actually not flat but a curved surface. For the shear band morphology near the side surface (Fig. 3b), it has been explained based on 2D stress field in many reports, which consider the shear direction to be parallel to the side surface^10^,^11^,^21^. However, the shear-offsets in our experiment actually suggest a shear direction almost perpendicular to the side surface. It seems that these present understandings might to some extent contradict with real case. Besides, to comprehensively understand the different shear band morphologies is another confusing question. In this study, we speculated that these shear band morphologies didn’t come from the conventional 2D notch tip stress field, but should be considered in a 3D situation.

### 3D stress field simulation by finite element method

**Figure 4:**
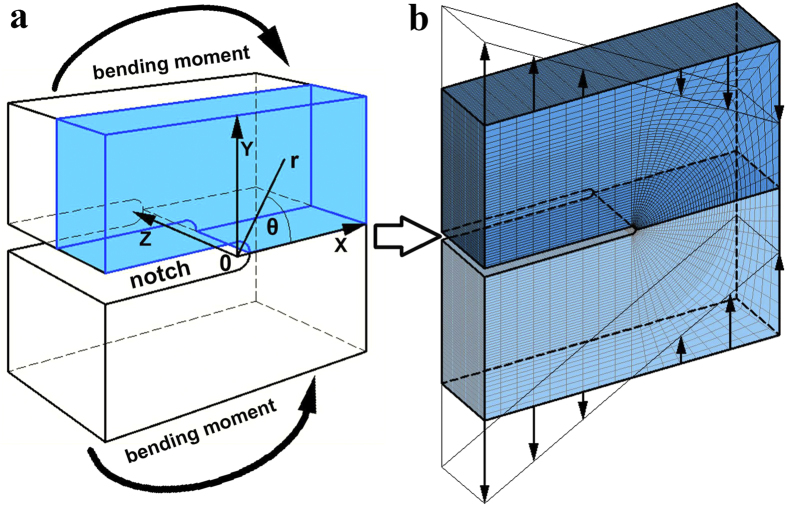
(**a**) Illustration of the FEM modeled sample with coordination system. (**b**) The element mesh in FEM simulation. According to the symmetry of the sample, only a quarter of the model was meshed, which was the light-blue part in (**a**). The regions marked with different letters in (**b**) used different element mesh.

For this aim, we performed a 3D notch tip stress field simulation by FEM. This method has already been applied to study the shear bands in metallic glasses^22^,^23^. It doesn’t aim to reveal the microscopic structure change inside the shear band^24^,^25^, but to provide macroscopic stress field information. Our model is shown in Fig. 4, which represents the notch tip region of a bar shape sample. To better represent the real case, the size of the model, including notch radius, ligament and thickness, and the external force, were all set based on the experiment. Details of the simulation are shown in Methods.

**Figure 5:**
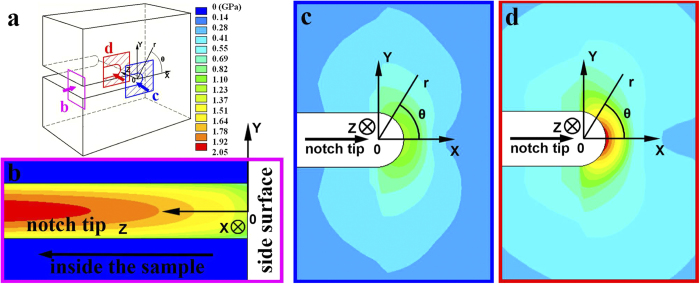
The finite element simulation result of the 3D shear stress distribution near the notch tip. The results are shown from three different perspectives as illustrated in (**a**) with different colors. (**b**) shows the stress distribution along the z-axis. (**c**) shows the stress distribution on the side surface. (**d**) shows the stress distribution inside the sample.

The simulated notch tip stress field distribution is shown in Fig. 5 from three perspectives. Figure 5b shows the shear stress distribution along the z-axis, from side surface to the center of the sample. It could be found that the stress increases when it goes deeper inside the sample from the side surface (larger z value). Figure 5c,d show the shear stress distribution in x-y plane on the side surface and in the center of the sample, respectively. It could be found that higher stress locates at a shorter distance from the crack tip (smaller *r* value) and a smaller angle from the original crack direction (smaller *θ* value).

This simulation result shows a mismatch between the stress intensity distribution and shear band morphology (Fig. 1). It indicates that the part of the material involved in plastic deformation doesn’t correspond to a unique stress value. Besides, the stress intensity is higher inside the sample (Fig. 5d) than that on the side surface (Fig. 5c). However, the shape of stress distribution hasn’t changed much, which cannot explain the different shear band morphologies on the side surface and inside the sample (Fig. 3). Moreover, the final fracture took place along the later formed shear band at *θ* ≈ 70°, where the stress intensity was relatively low, but not the initially formed shear band at *θ* ≈ 0° with higher stress intensity (Fig. 2). All these results, apparently, are inadequate to explain the as-observed shear band behavior.

The above fracture experiment and stress field simulation show a shear band controlled deformation behavior different from the conventional material and fracture mechanics. Many questions are still remained: (1) Does the yielding criterion exist in metallic glasses? Or why the shape of the shear bands and the plastic region did not follow the stress intensity distribution? (2) How could the shear band multiplication process be achieved at the notch tip of these work-softening materials? (3) Why the shear bands formed in a sequence manner from low angle part to high angle part? (4) Why the final fracture didn’t take place at the firstly formed shear band but the lately formed shear band with apparently low stress intensity? (5) Why the shear band morphologies on the side surface and inside the sample are different?

### Principle of shear band deformation

To resolve the above questions, the principle of shear band deformation and its difference from the classic materials mechanics should be reconsidered first. According to the plastic flow study in metallic glass^26^,^27^,^28^, the shear band could be regarded as a localization of shear transformation zone (STZ) in a ~20 nm thin layer, which could slide by viscous flow with a relatively low viscosity. The shear band movement could be generalized into four steps: nucleation, propagation, sliding and multiplication/fracture. The nucleation of shear band could be approximately related to a critical shear stress when the initial STZ events are activated^26^,^29^. With shear dilatation effect^19^,^30^, those sheared regions would become looser and softer, resulting in more STZs localized on the same plane and nucleation of shear bands. After that, the shear band would propagate quickly with more strain localized in front of the shear band tip. As many studies suggested^31^,^32^,^33^, this propagation direction was approximately parallel to the maximum shear stress direction. Then by viscous flow, the as-formed shear bands can slide and relax the local shear stress gradually. If multiple shear bands can be activated somehow before final failure, better plasticity is expected. Finally, the material might fracture along the shear band paths due to the significantly reduced local viscosity.

In this shear band controlled deformation process, it should be noticed that the physical meaning of the critical stress required to activate a shear band differs from the conventional yielding stress in crystalline metals. In conventional materials mechanics, the work-hardening effect will result in a continuous and homogeneous stress/strain field, where the material deforms only when the local stress reaches the yielding stress. In metallic glasses, although the Mohr–Coulomb criterion^34^ and elliptical criterion^35^ were proposed based on uniaxial compression and tension strength data, however, it should be noticed that these criteria actually described the stress state for shear band nucleation. After that, due to the work-softening effect, the deformation could spread at lower stress, which brings doubt on applying the conventional yielding concept in these materials. Here we speculated that in metallic glasses, the stress needed to sustain shear band propagation would be far below the critical stress for nucleation, due to the work-softening effect and the stress concentration at the sharp shear band front. It could then be expected that the shear band propagation path and therefore the deformed region of the material will follow the local shear stress direction but not the yielding stress distribution. With this understanding, we will reconsider the shear band morphology in our experiment based on shear stress direction instead of stress intensity distribution.

### Reproduction of shear band morphologies

To reproduce the shear band morphology, the shear stress distribution has been already achieved in our FEM simulation. The next matter is to determine the shear band nucleation point under this stress field. As discussed previously, the shear bands nucleate at a critical shear stress. For the Pd-Cu-Si BMG used in our experiment, the plastic deformation occurs at ~1370 MPa^36^, which expects a critical shear stress of less than 1 GPa. According to our FEM simulation based on the same experimental condition, the stress intensity at most parts of the notch tip on the side surface is beyond 1 GPa (Fig. 5c), and is even higher inside the sample (Fig. 5d). It suggests that all the notch tip at different angle *θ* could reach the critical stress and become potential shear band nucleation site. The experiment result (Fig. 1) also shows that shear bands indeed have formed from all around the notch tip region.

From these nucleation sites, the shear band morphology could then be determined based on the local shear stress direction. Thereby we could draw a series of shear band morphologies at different nucleation points at the notch tip. In Methods we have shown how this simulation and data processing could be achieved. Here we will first show two typical as-drawn shear band morphologies, from two nucleation points at *θ* = 70° on the side surface and inside the sample according to the as-observed fracture angle (Fig. 2h). (The formation of multiple shear bands at different angles would be discussed in the next section)

**Figure 6:**
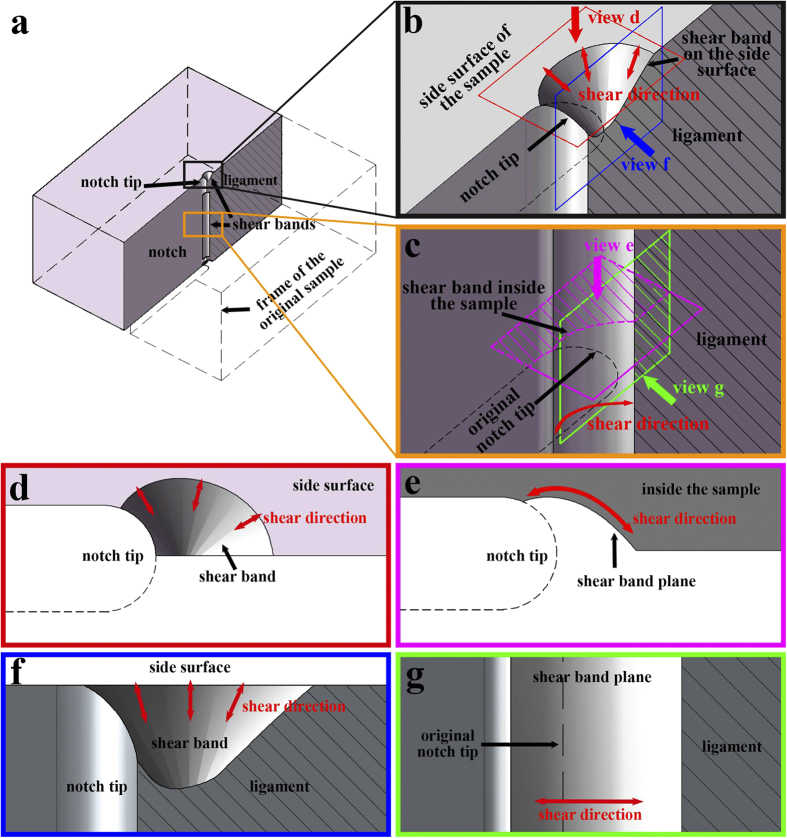
The results of the two different shear band morphologies determined by local maximun shear stress in the FEM simulated shear field. (**a**) shows the positions of the two shear bands. (**b**,**d**,**f**) show the shear band morphlogy near the side surface from three perspectives as shown in (**b**). (**c**,**e**,**g**) show the shear band morphlogy inside the sample from three perspectives as shown in (**c**).

Figure 6 shows the simulated shear bands from different perspectives. The two shear bands exhibit two different morphologies, which result from the different maximum shear stress directions at different positions of the notch. Inside the sample, the maximum shear stress direction is on the x-y plane, similar to that under a plane strain condition. As this direction alters continuously during shear band propagation, an arc-shaped shear band could form as shown in Fig. 6b,d,f. On the side surface, the stress distribution is different. The minimum principal stress is perpendicular to the side surface with zero intensity, and the other two principal stresses are both tensile and parallel to the side surface. Thus the direction of the maximum shear stress points to an angle of 45° from both the x-y plane and z-axis. Then a cone shape shear band could form as shown in Fig. 6c,e,g.

Our simulated shear band morphologies are remarkably consistent with the as-observed shear bands and fracture morphologies in Figs 2 and 3. The shear directions also agree with the as-observed shear-offsets. The results are also well supported by many other experimental reports of other ductile BMGs^10^,^11^,^20^,^37^.

### Reproduction of shear band multiplication process

With the shear band morphology being successfully explained, the next question is to understand the macroscopic deformation by multiple shear bands movement. The above experiment and simulated stress field results suggest that multiple shear bands could nucleate at different angles from the notch tip. To reproduce these shear bands, another simulation will be shown in the following.

**Figure 7:**
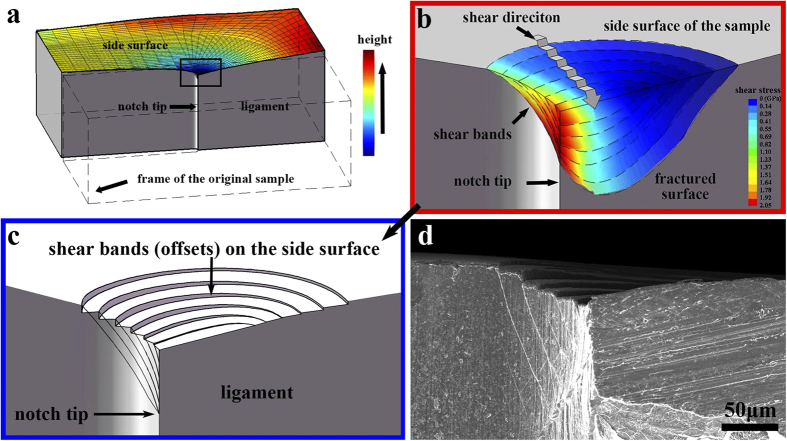
The simulated and experimental shear band morphologies with shear-offsets on the side surface of the sample. (**a**) The simulated multiple shear band morphologies at different angles from the notch. (**b**) The shrinking tendency on the side surface (before shear band formation) predicted by FEM simulation. (**c**) The simulated shear-offsets after shear band sliding by speculating that the shear-offsets size is accordant with the original shrinking extent. (**d**) An experimental crack tip morphology. The color bar in (**a**) represents the shear stress distribution on the shear band sites.The color bar in (**b**) represents the simulated height of the sample’s side surface under bending loading.

Now consider a series of initiating points on the side surface at angles ranging from 0° to 90° with a spacing of 15°. By the same method in Fig. 6, the morphologies of these shear bands could be simulated out and shown in Fig. 7a. It should be noticed that this setting is only to roughly represent the multiple shear bands but not a real distribution.

The result shows that all those shear bands have a similar cone shape. With an increasing nucleation angle *θ*, the shear band could propagate to a larger region. As the simulated stress field (Fig. 5) shows that high stress could be reached at small angle, it could then be reasonably expected that this location will reach the critical stress earlier than other places and form the initial shear bands. By sliding of these shear bands, the local shear stress near the shear band site will be relaxed, whereas the material outside the shear bands is still kept in elastic region. During further loading, the stress at higher angle of the notch tip will continue to increase and approach to the critical stress. Then new shear bands could nucleate at the high angle and propagate to larger area. By this process, more and more shear bands will form in a sequence manner from small angle to high angle with increasing size, and the plastic region of the material will also extend with the formation of those shear bands. By this understanding, the shear band multiplication in this fracture process could be reasonably understood. The main difference of this shear band controlled behavior from the conventional LEFM is, the plastic region follows the local shear stress direction, but not the yielding stress distribution. And the plasticity inside the region is heterogeneous due to the formation of separated shear bands instead of continuous deformation.

One of the results of this shear banding is the shear-offsets on the side surface, formed during shear band sliding. As mentioned earlier, the shear direction of these shear bands at the side surface is not along the x-y plane, but points to an angle of 45° from both the x-y plane and z-axis. This unique shear direction could not be explained by neither the plane strain nor plane stress model in a 2D case^6^,^11^,^18^. Under tensile stress, the material tends to shrink, which is similar to the necking effect in polycrystalline alloys. In our FEM simulation results, we found obvious shrinking near the notch tip, where the stress intensity is high (Fig. 7b). After the shear band formation, the side surface will become discontinuous and separated by shear-offsets.

To reproduce these shear-offsets, we speculated that the shape of the side surface after shear-offsets formation will be accordant with the original shrinking extent. Thereby the shear-offset size could be evaluated by the shrinking extent difference between adjacent shear band sites. From the given shear band distribution in Fig. 7a, the shear-offset morphology could then be obtained based on the shrinking extent distribution in Fig. 7b (details of this simulation are shown in Methods). The simulated result is shown in Fig. 7c. It excellently agrees with the real crack tip morphology as shown in Fig. 7d. However, it should also be noticed that as the shear band spacing were chosen manually, and the shrinking extent was based on the original elastic model, the shear-offset morphology in our simulation is only a rough estimation just to help understanding the shrinking effect and the shear-offsets distribution on the sample’s side surface. More precise simulation is still needed for further study.

In Fig. 7c, the shear bands at small angle *θ* form relatively small shear-offsets, and the shear bands at larger angle *θ* form larger shear-offsets. The reason is that with an increasing size, the shear band could bear more plastic deformation. With the shear dilatation effect^19^,^30^, these shear bands with larger sliding distance would become weaker than those formed earlier. Some studies also suggested that the shear band ruptured when it reached some critical shear-offset size. Thus the final fracture in experiment would occur along the later formed shear band at high angle with large shear-offset but not the early-formed shear bands.

Based on the above simulation and discussion, a general fracture and deformation process of metallic glasses could be depicted as the following four steps: (1) The material deforms elastically under low stress intensity. (2) The notch tip at low angle *θ* will first reach the critical stress and form shear bands along the maximum shear stress direction. (3) Then by sliding of the shear bands, the local shear stress at shear band site will be relaxed. But the stress outside the shear band region will continue to grow, and reach the critical stress at higher angle and form larger shear bands. The plastic region will also extend with the formation of new shear band. (4) Finally, fracture would occur along a relatively weak shear band. This fracture process could be clearly confirmed by the experiment (Figs 2 and 3 and the video in supplementary) and the FEM simulation.

## Discussion

Above we depicted a new fracture process in metallic glasses, which differs from the conventional “plastic zone” description in LEFM. In this process, the shear band morphology and the plastic region of the material follows the local shear stress direction but not the yielding stress distribution. And the deformation inside the plastic region is discontinuous due to separated shear bands. We found 3D stress field to be necessary in prediction such shear band morphology under complicated stress field condition. This method might also help to understand other situations such as composites, heterogeneous structure, near flaws, in complicate shape sample and under other complicate stress state.

Although our simulation fit well with experiment, we should notice that it is still an approximation, as it is based on pure elastic analysis. In the real case, the formation of shear bands could relax the local stress and lead to stress redistribution. This is also a common question for the LEFM in polycrystalline alloys, where the plastic deformation near the notch tip region will also redistribute the stress, making the real plastic zone deviate from the pure elastic description. By considering this stress relaxation effect in a continuous stress field, the Irwin’s model suggested the real plastic zone size to be much larger than that in the original elastic model^15^. And the Hutchinson-Rice-Rosengren (HRR) theory proposed another quantitative description of elastic–plastic notch tip field based on strain-hardening level^15^. However, the simplification of LEFM is still widely accepted, as it provides a more accessible method to understand the fracture process in polycrystalline alloys. In metallic glasses, the simulation in this study is also based on elastic analysis, which is similar to the LEFM in polycrystalline metals. Comparing to the experimental results, our simulation shows that the elastic analysis could well reproduce the shear band morphologies in experiments, probably because the stress redistribution actually would not change the shear stress direction very much. Therefore, the elastic model might be a reasonable simplification for describing the morphologies of shear band region or plastic region in metallic glasses. Inside the plastic region, it should be noticed that the stress field would redistribute by the shear band movements, and would become discontinuous at the shear band path. The exact stress field inside the plastic region cannot be directly represented by the FEM simulation. Modifications and new theoretical tools would be needed for further study of the discontinuous stress field inside the plastic region.

Before our study, there were some misunderstandings on the shear band morphology in BMG fracture. To interpret the cone shape shear band morphology on the side surface, some studies used the Prandtl slip line field theory^6^, which also considered that the shear band would propagate along the maximum shear stress direction and could draw curved shear band paths. Some studies considered the mode II or a mixed-mode stress field distribution^11^,^18^. However, those studies ran their simulations all based on the 2D crack tip stress field and expected a shear direction parallel to the side surface. According to our results in Figs 1, 2, 3, we showed that the formation of shear band morphology was actually a 3D case. Especially, the situation on the side surface was totally different from the ideal 2D plane stress/strain condition. To interpret the shear band morphology inside the sample, some studies induced the concept of “flat zone”^19^,^20^ according to the fractograph under SEM, but there was a lack of explanation on why this zone was flat. According to our experiment in Fig. 3 and simulation in Fig. 6, we showed that the “flat zone” was actually not flat but a curved surface. The above studies also faced difficulty in comprehensively interpreting the different shear bands inside the sample and on the side surface. In our study we revealed that these different shear band morphologies are just the results of the 3D distribution of maximum shear stress direction at different positions of the sample. Our study not only clarified those misunderstandings on shear direction and shear band morphology, but also provided a new insight to understand the deformation in metallic glasses. More importantly, it showed that the shear band controlled deformation will result in different concepts of “yielding stress” and “plastic zone” from the classic material and fracture mechanics.

To further study the mechanical behavior of these shear band controlled materials, we suggest that it could be considered in a new framework of multiple shear band deformation mechanism as used in our study. In such deformation, the material could deform at a low stress intensity after shear band nucleation. It also will be a dynamic process, with the overall deformation achieved step by step with individual shear band nucleation, propagation and sliding instead of continuous deformation. As the shear band behavior could be affected by the local stress distribution, the mechanical properties of the material will change accordingly with the loading condition. For instance, when the size and shape of the sample are changed, the morphology and shear-offset size of the shear bands will also change and lead to different deformation results. Therefore those mechanical properties including plasticity and fracture toughness, will strongly depend on both the material and external affections on stress condition. This all agree with experiments^6^,^7^,^8^,^9^. By this understanding, it becomes doubtful to treat those mechanical properties in these materials as intrinsic material properties. We hope that start from this work, new experimental methods, theoretical criteria, or definitions could be proposed to further study these shear band controlled materials, especially for the engineering important metallic glasses.

## Methods

### Sample fabricating and 3-point bend testing

The Pd_77.5_Cu_6_Si_16.5_ metallic glass, a typical ductile BMG with considerable glass forming ability, was selected in this work. The alloy ingot was fabricated by mixing the highly pure Pd (>99.99%), Cu (>99.99%) and Si plates (>99.99%) according to their atomic ratio and melted in a quartz tube under the protection of high purity Argon gas. Then the ingot was purified by fluxing method. B_2_O_3_ were melted and toasted in a quartz tube under a vacuum state of 10^−2 ^Pa and a temperature up to 1150 °C for more than 2 hours. After the melt became transparent and clear, the ingot was immersed into the melt under the same vacuum state and held for 4 hours. Such purification process was carried out for at least 3 times. Finally, a spherical sample was obtained by water quenching method. The sample was then cut and polished into a cuboid shape of 3 × 6 × 30 mm^3^. A notch with a width of 200 μm and length of 1.5 mm was introduced into the sample by using diamond wire saw. Then the sample experienced a 3-point bending test under a loading rate of 0.1 mm/s on a DL100 testing machine. An optical camera captured the evolution of shear-offsets on the sample’s side surface during the test. A LEO-1530 field emission scanning electron microscope (FE-SEM) was used to inspect the fractograph. After cutting the fractured piece from the middle, the cracking path inside the sample was explored by the SEM.

### Finite element method simulation

A 3D FEM model was employed in our study to simulate the notch tip stress field distribution in our sample. The model’s size was set to be 3 × 6 × 6 mm^3^ based on our sample for bending test and shown in Fig. 4. The modeled notch tip was set to be cylindrical with a radius of r = 0.1mm to fit the experimental condition. According to the symmetry of the sample, we only meshed a quarter of the model as shown in Fig. 4b. Finer mesh was used near the notch tip region. For details of the mesh, the elements in front of the notch tip (A zone) were set to a fan shape, with an angle spacing of 10°, and a radius spacing of 1.9/36 mm. The elements beside the notch tip (B zone) were set to a size of 2/9 × 1.9/36 mm. The elements next to them in C zone were set to a shape of 2/9 × 1.9/10 mm. The elements in D zone connected the fan shape zone with the outer boundary of the model. The boundary was equally divided by 9 and directly connected to the nodes on the outer boundary of the fan shape zone. This part was meshed into a 9 × 18 net. The model on its thickness direction (z-axis) was equally divided by 36. Finally, a finite element mesh with a total element number of 44712 and node number of 48692 was achieved as shown in Fig. 4b. According to the yielding load of the sample in 3-piont bending test, it corresponds to a bending moment of 10 N·m, equivalent to a stress intensity of K_Q_ ≈ 80MPa·m^0.5^. To approach this real case in our model, we applied a force with a uniform gradient onto the nodes on the upper bound of the model (Fig. 4b), and the total bending moment of the force equaled to 10 N·m. The nodes on the x-z plane in front of the notch were fixed on the plane for the symmetry of the sample. We performed the simulation in the ANSYS program with linear elastic elements (SOLID185). The elastic properties of the elements were set as E = 88.8 GPa and υ = 0.41, from the Pd_77.5_Cu_6_Si_16.5 _BMG data^38^. Then by static analysis, a 3D notch tip stress field and displacement distribution was achieved.

### Shear band morphology simulation

**Figure 8:**
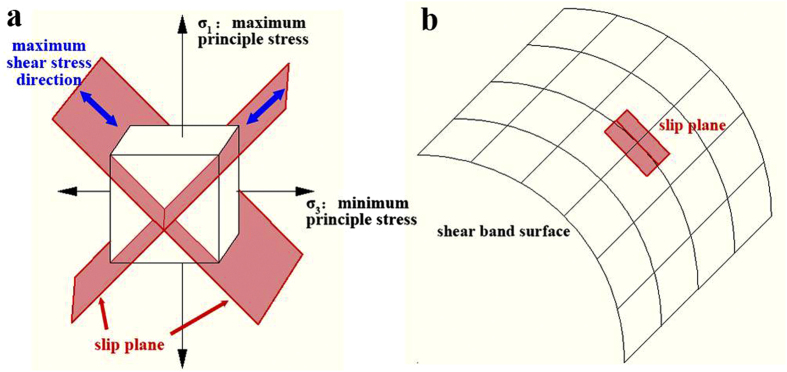
Illustration of shear band morphology determination. (**a**) When the directions of the three principle stresses at a certain point are given, two slip planes could be determined along the maximum shear stress direction. (**b**) From a given nucleation point, we could step by step draw a 3D surface, on which the tangent plane at any point parallels to the local slip plane.

Here we will generally explain the method we used to obtain the shear band morphology from a given nucleation point in our simulated stress field. The 3D FEM simulation provides the original stress distribution. To simulate the shear band morphology, we needs to carry out the data processing outside the FEM program. Firstly, the matrix which included the stress, strain and displacement data on each node of the meshed model during loading was exported from the FEM program. The matrix operation can be easily proceeded in a self-create Matlab script, and the maximum shear stress direction could then be determined along the angular bisector between the maximum and minimum principle stresses (Fig. 8a). We notice that there are two maximum shear stress direction on each node, and are perpendicular to each other. In a 3D case, if we choose one of them as a normal vector, we could obtain a plane (here we call it as slip plane) parallel to the other maximum shear stress direction. In a complicate stress field like the notch tip, technically, if we draw a surface, on which the tangent plane at any point parallels to the local slip plane, the whole surface would be along the shear stress direction and represent the shear band morphology (Fig. 8b).

To draw such surface, the location of nucleation point should be considered first. In our 3D stress field simulation we showed that the nucleation points could locate at different angles on the notch tip from both the side surface and inside the sample. As the shear stress directions on the surface and inside the sample are different, they should be considered separately.

From a nucleation point on the side surface, based on the stress at the point, the local shear stress direction (which points into the sample) and local slip plane coordinate (its normal vector is the other shear stress direction) could be achieved. Then we could calculate out the coordinate of the intersection line between the slip plane and the sample’s side surface. Along this intersection line we could determine another point next to the nucleation point, with a small step length of 5 μm. Then for the new point, the local stress condition will change accordingly, by which a new slip plane could be determined. Then we could determine the next point on the new intersection line. Following the same procedure, a series of points could be determined. By connecting these points, a curved line that represents the intersection between the curved shear band plane and the sample’s side surface, could be obtained. From each of these as-obtained points on the side surface, we could then step by step draw more points along the local maximum shear stress directions (pointing into the sample) until reach the edge of the model. Finally these points formed a grid. By connecting these points, a 3D surface could be obtained as shown in Fig. 6. As we expected, it could represent the shear band morphology from this given nucleation point.

From a nucleation point inside the sample, based on the stress at the point, the local shear stress direction (which is on the x-y plane) and local slip plane coordinate could be achieved. We could then similarly determine a series of points along the intersection line between the local slip plane and the notch tip surface. Then from these points, we could determine more points along the local maximum shear stress direction, with a step length of 10 μm. Finally these points formed a grid. By connecting these points, another 3D surface was obtained as shown in Fig. 6. We expected that this surface could represent shear band morphology from this given nucleation point.

By this method, the shear band morphologies from different nucleation points of the notch tip could be achieved.

### Shear-offset morphology simulation

With the formation of shear bands, the sample’s side surface would be separated into several sub-planes. Here for the given shear band distribution (Fig. 7a), we numbered the sub-planes from 1 to 6 according to their position. The boundary of the sub-planes could be determined by the intersection lines between the shear bands and the sample’s surface. To estimate the shear-offset size, we speculated that after shear bands formation, the shrinkage of each sub-plane would be still accordant with the original shrinking extent in the elastic model (Fig. 7b). Then we could obtain the z-coordinate at each point of the intersection lines based on the shrinking extent in the elastic model. As each intersection line is on a sub-plane, we could then evaluate the position of the sub-plane based on the intersection line coordinate. And we found that after shrinking, the curved intersection lines will be still approximately in planes. Then based on three points on the intersection line (here we chose the nucleation point, the point at y = 0 and the point in the middle of the intersection line as marked by the red dots in Fig. 7c), we could achieve the plane coordinate, which as we expected, could approximately represent the sub-plane related to the intersection line. Thereby we achieved the coordinates of the six sub-planes from the six shear bands in Fig. 7a. The different shrinking extents between the sub-planes will form gaps, which represent the size of shear-offsets. Finally by connecting these sub-planes together we achieved the shear-offsets morphology as shown in Fig. 7c.

## Additional Information

**How to cite this article**: Yang, G. N. *et al.* The shear band controlled deformation in metallic glass: a perspective from fracture. *Sci. Rep.* **6**, 21852; doi: 10.1038/srep21852 (2016).

## Supplementary Material

Supplementary Video

## References

[b1] Greer A. L., Cheng Y. Q. & Ma E. Shear bands in metallic glasses. Mater. Sci. Eng., R 74, 71–132 (2013).

[b2] Hu N. & Molinari J. F. Shear bands in dense metallic granular materials. J. Mech. Phys. Solids. 52, 499–531 (2004).

[b3] Wei Q., Jia D., Ramesh K. T. & Ma E. Evolution and microstructure of shear bands in nanostructured Fe. Appl. Phys. Lett. 81, 1240–1242 (2002).

[b4] Olmsted P. D. Perspectives on shear banding in complex fluids. Rheol. Acta 47, 283–300 (2008).

[b5] Zhang H. W., Maiti S. & Subhash G. Evolution of shear bands in bulk metallic glasses under dynamic loading. J. Mech. Phys. Solids. 56, 2171–2187 (2008).

[b6] Hassan H. A., Kecskes L. & Lewandowski J. Effects of changes in test temperature and loading conditions on fracture toughness of a zr-based bulk metallic glass. Metall. Mater. Trans. A 39, 2077–2085 (2008).

[b7] Lewandowski J. J. Effects of annealing and changes in stress state on fracture toughness of bulk metallic glass. Mater. Trans. 42, 633–637 (2001).

[b8] Fujita K. *et al.* Effects of loading rates, notch root radius and specimen thickness on fracture toughness in bulk metallic glasses. J. Alloy. Compd. 434, 22–27 (2007).

[b9] Narayan R. L., Tandaiya P., Garrett G. R., Demetriou M. D. & Ramamurty U. On the variability in fracture toughness of ‘ductile’ bulk metallic glasses. Scripta Mater. 102, 75–78 (2015).

[b10] Demetriou M. D. *et al.* A damage-tolerant glass. Nat. Mater. 10, 123–128 (2011). 21217693 10.1038/nmat2930

[b11] He Q., Shang J. K., Ma E. & Xu J. Crack-resistance curve of a Zr–Ti–Cu–Al bulk metallic glass with extraordinary fracture toughness. Acta Mater. 60, 4940–4949 (2012).

[b12] Lewandowski J. J., Gu X. J., Shamimi Nouri A., Poon S. J. & Shiflet G. J. Tough Fe-based bulk metallic glasses. Appl. Phys. Lett. 92, 091918 (2008).

[b13] Lowhaphandu P. & Lewandowski J. J. Fracture toughness and notched toughness of bulk amorphous alloy: Zr-Ti-Ni-Cu-Be. Scripta Mater. 38, 1811–1817 (1998).

[b14] Xu J., Ramamurty U. & Ma E. The fracture toughness of bulk metallic glasses. JOM 62, 10–18 (2010).

[b15] Anderson T. L. Fracture mechanics: fundamentals and applications (Taylor & Francis, 2011).

[b16] Vaidyanathan R., Dao M., Ravichandran G. & Suresh S. Study of mechanical deformation in bulk metallic glass through instrumented indentation. Acta Mater. 49, 3781–3789 (2001).

[b17] Tandaiya P., Ramamurty U. & Narasimhan R. Mixed mode (I and II) crack tip fields in bulk metallic glasses. J. Mech. Phys. Solids. 57, 1880–1897 (2009).

[b18] Tandaiya P., Narasimhan R. & Ramamurty U. On the mechanism and the length scales involved in the ductile fracture of a bulk metallic glass. Acta Mater. 61, 1558–1570 (2013).

[b19] Gu X. J., Poon S. J., Shiflet G. J. & Lewandowski J. J. Ductile-to-brittle transition in a Ti-based bulk metallic glass. Scripta Mater. 60, 1027–1030 (2009).

[b20] Lowhaphandu P., Ludrosky L. A., Montgomery S. L. & Lewandowski J. J. Deformation and fracture toughness of a bulk amorphous Zr–Ti–Ni–Cu–Be alloy. Intermetallics. 8, 487–492 (2000).

[b21] Flores K. M. & Dauskardt R. H. Enhanced toughness due to stable crack tip damage zones in bulk metallic glass. Scripta materialia 41, 937–943 (1999).

[b22] Wang S. G., Sun M. Y., Song Z. Q. & Xu J. Cast defects induced sample-size dependency on compressive strength and fracture toughness of Mg–Cu–Ag–Gd bulk metallic glass. Intermetallics. 29, 123–132 (2012).

[b23] Zhao M. & Li M. Interpreting the change in shear band inclination angle in metallic glasses. Appl. Phys. Lett. 93, 241906 (2008).

[b24] Shao Y., Yang G. N. & Yao K. F. Nanocrystalline Phase Formation inside Shear Bands of Pd-Cu-Si Metallic Glass. Adv. Mater. Sci. Eng. 2014, 490181 (2014).

[b25] Shao Y., Yao K. F., Li M. & Liu X. Two-zone heterogeneous structure within shear bands of a bulk metallic glass. Appl. Phys. Lett. 103, 171901 (2013).

[b26] Argon A. S. Plastic deformation in metallic glasses. Acta Metall. 27, 47–58 (1979).

[b27] Spaepen F. A microscopic mechanism for steady state inhomogeneous flow in metallic glasses. Acta Metall. 25, 407–415 (1977).

[b28] Eastgate L. O., Langer J. S. & Pechenik L. Dynamics of large-scale plastic deformation and the necking instability in amorphous solids. Phys. Rev. Lett. 90, 045506 (2003). 12570434 10.1103/PhysRevLett.90.045506

[b29] Schuh C. A., Hufnagel T. C. & Ramamurty U. Mechanical behavior of amorphous alloys. Acta Mater. 55, 4067–4109 (2007).

[b30] Sun B. A. *et al.* Serrated flow and stick–slip deformation dynamics in the presence of shear-band interactions for a Zr-based metallic glass. Acta Mater. 60, 4160–4171 (2012).

[b31] Leamy H. J., Wang T. T. & Chen H. S. Plastic flow and fracture of metallic glass. Metall. Trans. 3, 699–708 (1972).

[b32] Hofmann D. C. *et al.* Designing metallic glass matrix composites with high toughness and tensile ductility. Nature 451, 1085–U1083 (2008). 18305540 10.1038/nature06598

[b33] Tay T. E., Yap C. M. & Tay C. J. Crack tip and notch tip plastic zone size measurement by the laser speckle technique. Eng. Fract. Mech. 52, 879–893 (1995).

[b34] Schuh C. A. & Lund A. C. Atomistic basis for the plastic yield criterion of metallic glass. Nat. Mater. 2, 449–452 (2003). 12792648 10.1038/nmat918

[b35] Chen Y., Jiang M. Q., Wei Y. J. & Dai L. H. Failure criterion for metallic glasses. Philos. Mag. 91, 4536–4554 (2011).

[b36] Yao K. F., Yang Y. Q. & Chen N. Mechanical properties of Pd-Cu-Si bulk metallic glass. Intermetallics. 15, 639–643 (2007).

[b37] Garrett G. R., Demetriou M. D., Chen J. & Johnson W. L. Effect of microalloying on the toughness of metallic glasses. Appl. Phys. Lett. 101, 241913 (2012).

[b38] Lewandowski J. J., Wang W. H. & Greer A. L. Intrinsic plasticity or brittleness of metallic glasses. Phil. Mag. Lett. 85, 77–87 (2005).

